# A Review of Button Battery Ingestions in Children—Diagnosis and Management

**DOI:** 10.3390/children12121678

**Published:** 2025-12-10

**Authors:** John Amodio, Michelle Lightman

**Affiliations:** 1 Children’s Hospital of Montefiore, Bronx, NY 10467, USA; 2 Albert Einstein College of Medicine, Bronx, NY 10461, USA; michelle.lightman@einsteinmed.edu

**Keywords:** ingestion of button battery, children, imaging

## Abstract

The production and use of button batteries (BBs) has gradually increased over the decades and has become commonplace in today’s world. As more household products have been using this type of battery, ingestions of these objects continue to rise. Over 83,000 battery ingestions in children were reported by the National Poison Data System between the years of 1985 and 2017. Over 77% of these were children less than 6 years of age. Between 1999 and 2019, the United States National Poison Data System reported a 66.7% increase in yearly ingestion of button batteries (6.98 to 10.46 per million population) and a 10-fold increase in complications. In this article, we review the epidemiology, mechanism of injuries to the esophagus and surrounding structures, complications detected with imaging, and management of button battery ingestions in the pediatric age group.

## 1. Introduction

In 1945, Ruben and Mallory founded the company Duracell, which produced the first zinc mercuric oxide alkaline button battery. As production of these batteries continues to rise, more household products have been using this type of battery, causing ingestions of these objects to rise as well. Between the years of 1985 and 2017, the National Poison Data System reported over 83,000 battery ingestions in children. Over 77% of these were children less than 6 years old. Between 1999 and 2019, the United States National Poison Data System reported a 66.7% increase in yearly ingestion of button batteries (6.98 to 10.46 per million population) and a 10-fold increase in complications [1]. According to Litovitz, Whitiker, and Clark, this can be attributed to the now commonly made and used 20 mm (and other large-sized) button batteries [2].

The most common source of button battery ingestions is from household products. According to Lorenzo, approximately 62% of BBs ingested come from household products, such as hearing aids, toys, and remote controls, while approximately 30% are discarded batteries and slightly less than 10% are taken directly from packaging [3]. Sethia, Gibbs, and Jacobs also point out that these ingestions may also source batteries from toothbrushes, lighted shoes, booklights, and thermometers [4].

### 1.1. Epidemiology

Battery ingestions are more common in younger children, and the associated complications are more frequent in children less than 5 years old [2]. Of particular interest is the BB with a 20 mm diameter. It is a very common size that also has a higher voltage than smaller BBs. Due to their size, these BBs can easily lodge in the esophagus of small children, similarly to other foreign bodies. However, Jatana, Barrona, and Jacobs point out that even smaller BBs, even as small as 12.5 mm, have been found lodged in the esophagus of very young children with no underlying esophageal pathology [5]. This is because both large and small batteries have the capacity to cause corrosive injury to the mucosa of the esophagus once ingested.

New button batteries are more likely to cause tissue damage when compared with old ones. However, it has been shown that even as little as a residual charge of 1.2 volts in any button battery can cause tissue damage [5]. Spent, used, or “dead” batteries can still cause significant tissue damage if swallowed and should be properly discarded.

**Figure 1:**
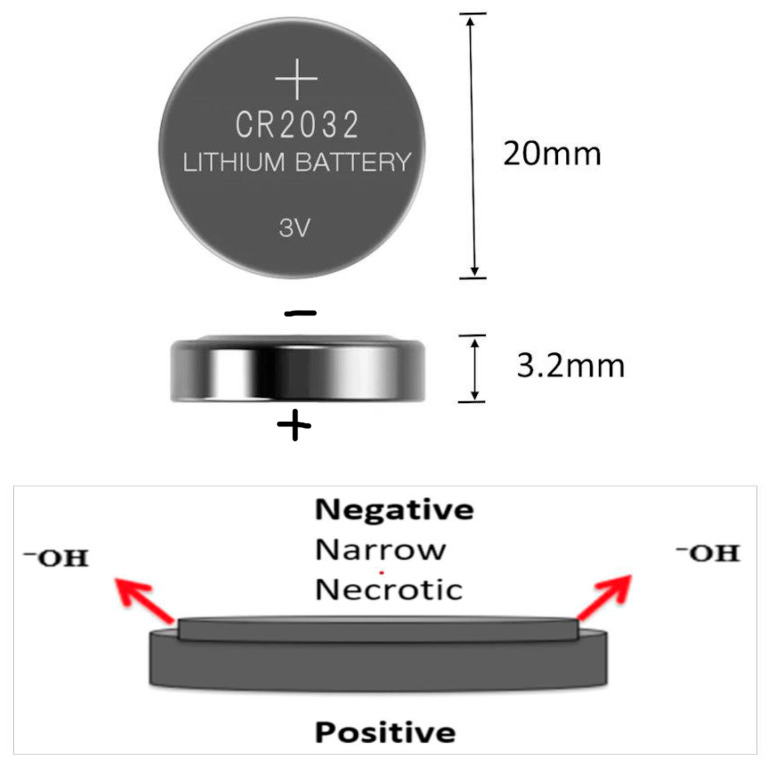
Anatomy of a button battery. The smaller ring is the anode, or negative pole. The larger ring is the cathode, or positive pole. The annotation “2032” stands for 20 mm diameter and 3.2 mm thickness.

Identifying the negative pole (Figure 1) of the battery is important, as it is the side of the battery that causes the most significant tissue damage. Once the negative pole is identified, the structures it is aligned with are then noted to be at higher risk of injury and complications and are therefore monitored more closely. This effect is due to the hydroxide ions that are emitted from the negative pole, which are thought to be the most detrimental to human tissues [6].

A mnemonic entitled the three Ns was crafted to help clinicians remember where to look for the most damage when identifying the button battery. The three Ns stand for negative–narrow–necrotic, which refers to the negative pole of the battery. This corresponds to the narrowest side of the battery on a lateral chest radiograph, and this negative and narrow side is what causes the most severe necrotic injury to the mucosa.

### 1.2. Mechanism of Injury

Injury to the affected tissue can be the result of one of three different mechanisms:

1. Electrical current at the mucosa: As the battery lies against the mucosa, there is a quick increase in pH caused by hydrolysis of water. The negative pole then generates hydroxide ions, which cause tissue necrosis. This is the most important mechanism of the three, with the remaining two thought to be of little importance.
2. Direct pressure on the mucosa.
3. Chemical leakage.

The most common button batteries are 3V; despite their lower voltage these batteries can also cause significant damage, albeit more slowly. Button batteries larger than 20 mm in diameter may cause even greater damage or complications since the larger size contributes to both a larger stored voltage and a decreased ability to pass through the esophagus without lodging there. Litovitz et al. [7] reviewed more than 60,000 cases of battery ingestions and showed that the batteries most associated with damage and injury were those with a diameter larger than 20 mm. Ingestion of these larger batteries was associated with 92.1% of major or fatal complications [7].

Within fifteen minutes of ingestion, button batteries are already able to produce visible injury to the mucosa. Within as early as two hours of ingestion, more serious injury and complications can begin to occur [8]. Some of these more serious injuries include esophageal ulceration and perforation.

Many factors and properties of the button batteries and of the patient contribute to the ultimate damage that the battery causes, which include the duration and location of the impaction; the size, orientation, and voltage of the BB; the size of the esophagus; and any potential underlying esophageal pathology of the patient.

### 1.3. Presentation

The signs and symptoms of button battery ingestion can vary and be vague. They may be confused with viral, respiratory, or gastrointestinal symptoms. The most severe outcomes typically arise in unwitnessed ingestions. This is because patients may be asymptomatic until a complication occurs. Some of the presenting signs or symptoms include cough, dysphagia, fever, vomiting, difficulty feeding, irritability, anorexia, dyspnea, and drooling [9,10]. Children who have lodged smaller batteries in the nasal cavity may present with nasal discharge, facial swelling, and periorbital cellulitis. Impacted batteries in the ear canal may produce a foul ear discharge if infected. Higher esophageal impactions, which tend to be more common in younger children, are more likely to result in feeding and respiratory difficulties. Mid-to-lower esophageal impactions are more common in older children and often result in pain and feeding and respiratory difficulties [11,12].

### 1.4. Immediate Treatment

If there is a known ingestion of a button battery, honey or sucralfate can be given on the way to the hospital to neutralize the alkaline environment set up by the battery [12,13]. It should be noted that honey is associated with an increased risk of botulism in infants [13].

The management of button batteries past the gastroesophageal junction is debated. According to some reports, complications from gastric batteries are rare, with only 7% and 1.3% occurring within the stomach or bowel, respectively [12]. A large Turkish study also noted that batteries that had passed through the pyloric sphincter continued to pass through the remaining GI tract spontaneously [14].

**Figure 2:**
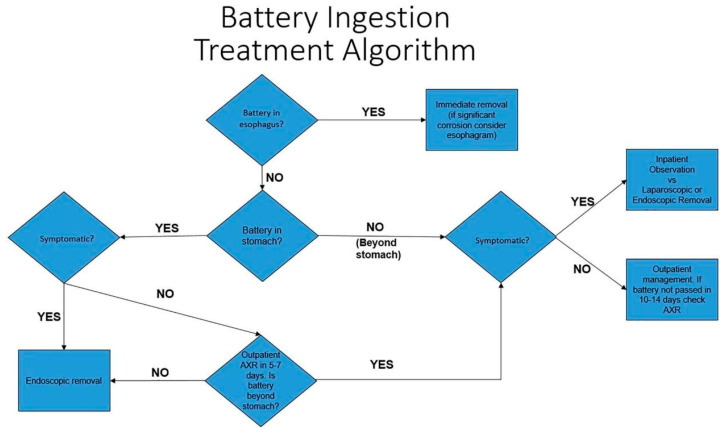
The NASPGHAN (North American Society for Pediatric Gastroenterology, Hepatology and Nutrition) Battery Ingestion Treatment Algorithm. Reprinted with permission from Rosenfeld, E.H., Sola, R., Yu. Y., St. Peter, S.D., & Shah, S.R. (2018). Battery ingestions in children: Variations in care and development of a clinical algorithm. *Journal of Pediatric Surgery,* 53(8), 1537–1541. https://doi.org/10.1016/j.jpedsurg.2018.01.017.

There are several care algorithms for button battery ingestions, as there are multiple clinical pathways due to the multidisciplinary nature of these injuries. The NASPGHAN (North American Society for Pediatric Gastroenterology, Hepatology, and Nutrition) recommends removal of the button battery immediately with endoscopy and conservative management of batteries past the gastroesophageal junction in asymptomatic patients [15]. This society has produced a management flow chart for button battery ingestions (Figure 2). According to multidisciplinary German guidelines, there is a benefit to performing an endoscopy in all cases of button batteries, even those that have already passed into the stomach [16]. This recommendation is provided in order to identify any esophageal injuries after passage of the battery and to remove any batteries found within the stomach. These guidelines recommend that all patients with a known battery ingestion should be kept fasting on arrival to the hospital in order for prompt endoscopic removal of all batteries found within both the esophagus and the stomach of the patient [16].

### 1.5. Diagnosis

**Figure 3:**
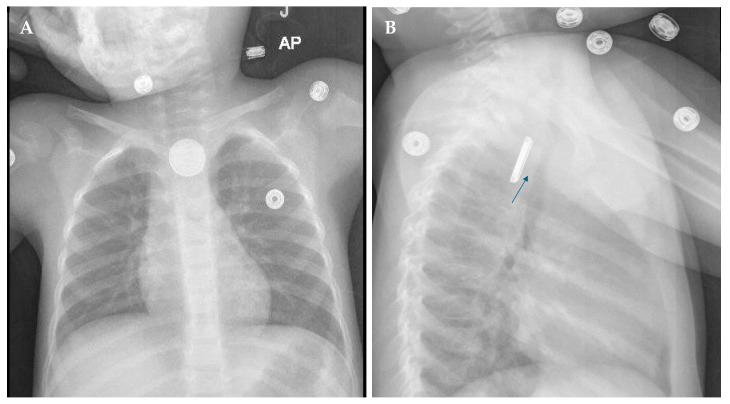
Frontal (**A**) and lateral (**B**) radiograph projections of the chest show an impacted button battery in the upper esophagus. The battery can be identified by the double-ring configuration. The smaller ring represents the anode. Note the separation of the trachea from the esophagus (arrow) due to esophageal edema.

The first step is conducting imaging to identify the location, size, and orientation of the button battery. This is performed with traditional radiographic examination. Both a frontal and lateral view of the chest should be imaged in all patients suspected of a button battery ingestion; however, additional views of the neck and abdomen may be necessary if the battery is not visualized on the chest imaging. On all imaging, the button battery has a double ring or halo appearance, as in Figure 3.

The frontal chest view may be more accurate than the lateral view, as some slimmer batteries may not have the well-known and identified “step-off appearance”. Another issue with the lateral view is that sometimes the images taken may not be perpendicular to the battery and thus are difficult to evaluate.

It is important to note the position of the smaller ring, or anode, as this orientation may alert the radiologist and the clinical staff to what complications may occur.

**Figure 4:**
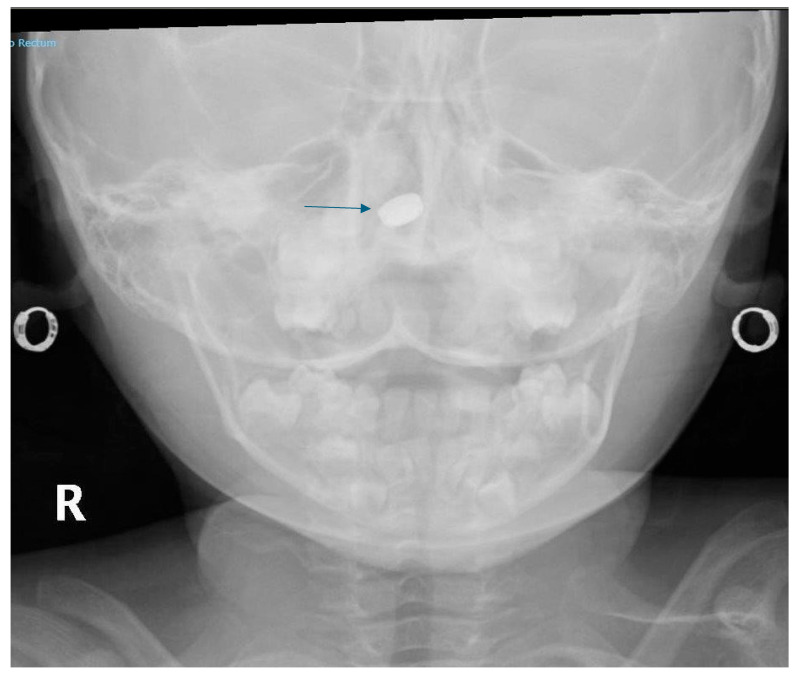
Small button battery impacted in right nasal cavity (arrow) on AP projection of the skull region.

As mentioned previously, smaller batteries may become lodged in the nasal cavity (Figure 4) and can result in nasal septal perforation, facial swelling, and periorbital cellulitis. If insertion into the nasal cavity or ear is suspected, AP and lateral views of the facial bones would be helpful.

Interestingly, there has been some research conducted using AI to distinguish button batteries from coins; however, more data sets are required before an absolute distinction can be made [17].

### 1.6. Imaging of Complications Secondary to Button Battery Ingestions

**Figure 5:**
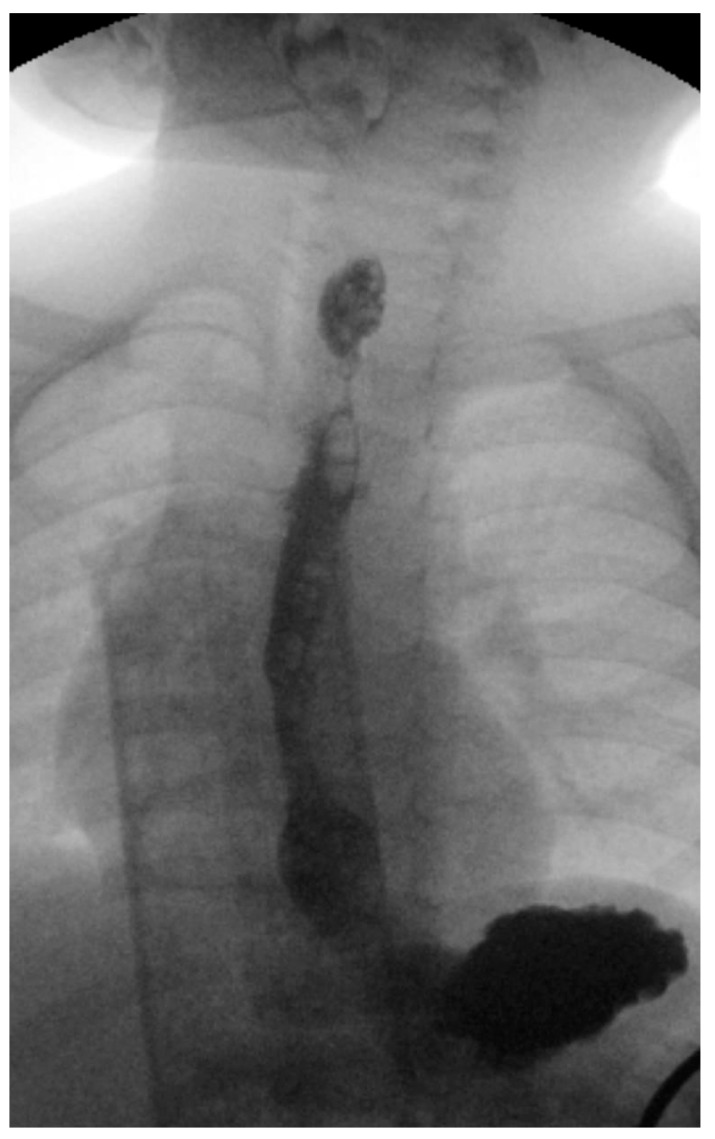
Radiograph highlighting esophageal stenosis (in the proximal esophagus several weeks after button battery removal. The patient had an aversion to liquids and solids.

**Figure 6:**
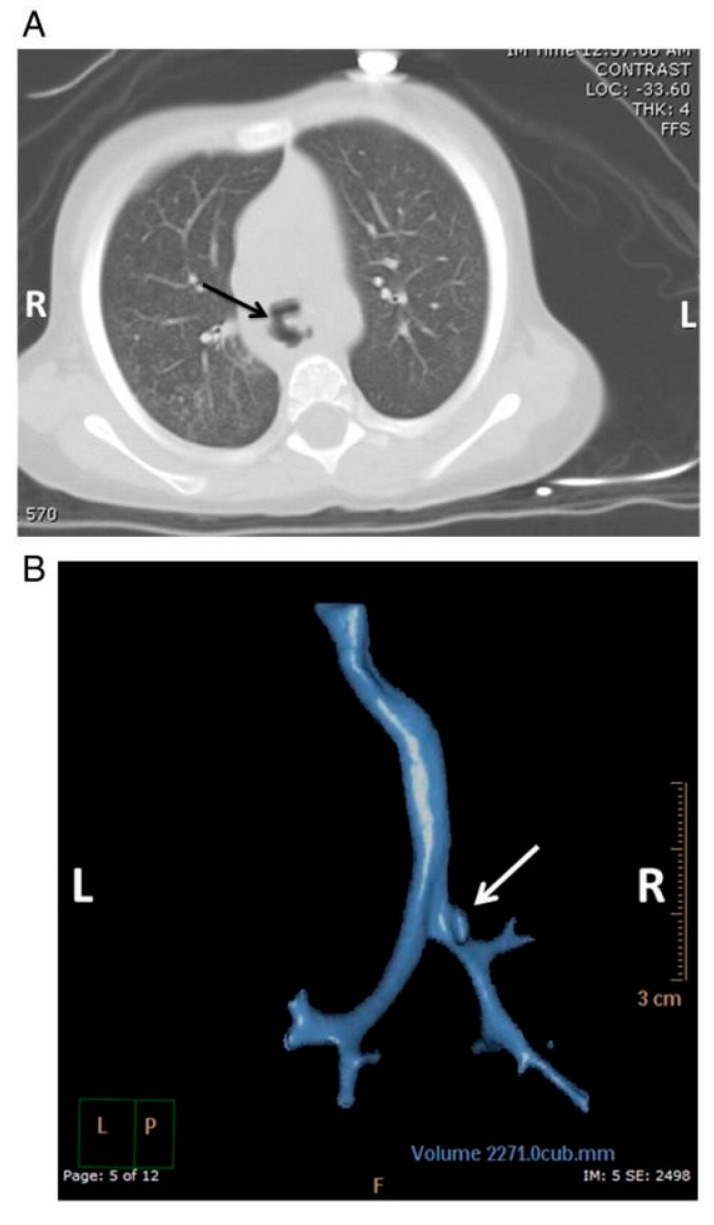
Axial CT scan (**A**) shows the connection of the esophagus and trachea (a tracheoesophageal fistula) after removal of a button battery. (**B**) A 3D reconstruction of the trachea shows the connection of a fistula with the mainstem bronchus (white arrow). Reprinted with the permission of Robert Russell, Mervyn Cohen, and Deborah F. Billmire. Tracheoesophageal fistula following button battery ingestion: Successful non-operative management *JPed Surg* Volume 48, Issue 2, p441–444, February 2013.

**Figure 7:**
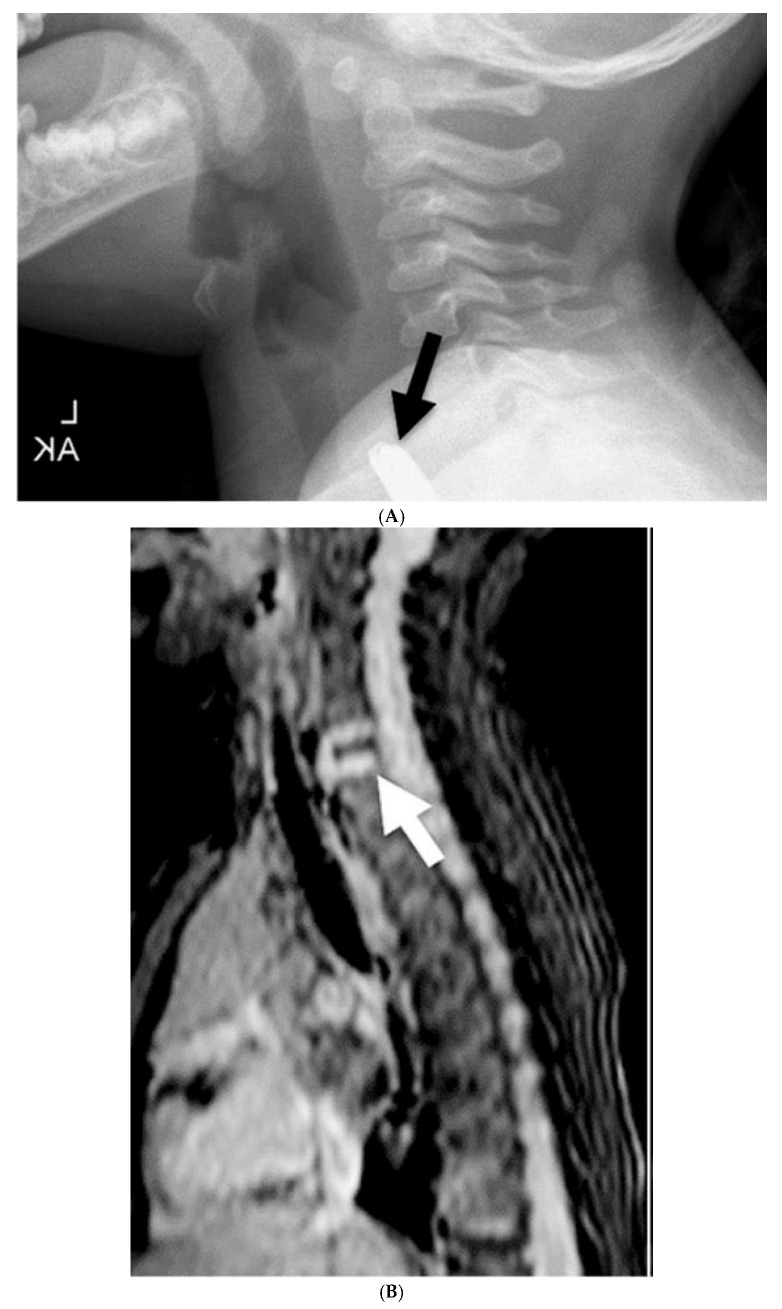
Lateral view of the neck (**A**) demonstrates an impacted button battery in the lower esophagus. Note: the anode is pointing posteriorly. Post-contrast MRI of the same region (**B**) demonstrates contrast enhancement of the discs at the site of prior BB impaction, which was compatible with spondylodiscitis. Reprinted with permission from Neil E.O. Grey, LaDonna J. Malone, Angie L. Miller, et al. Magnetic resonance imaging findings following button battery ingestion. *Pediatric Radiology*, 2021, 51:1856–1866 [20].

**Figure 8:**
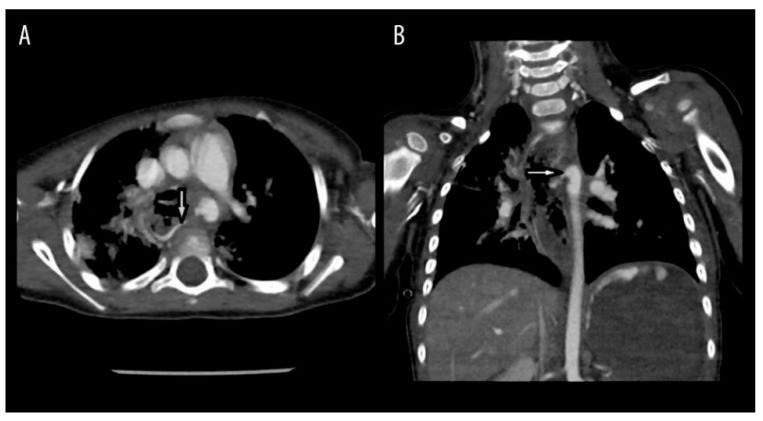
(**A**) Axial (**B**) Coronal A CTA of the chest demonstrated an aortoesophageal fistula (white arrow). Note the swallowed contrast in the stomach from the aorta–esophageal fistula. Reprinted with permission from Khalid M. Alreheili, Mansour Almutairi, and Ali Alsaadi. A 2-Year-Old Boy Who Developed an Aortoesophageal Fistula After Swallowing a Button Battery, Managed Using a Novel Procedure with Vascular Plug Device as a Bridge to Definitive Surgical Repair. *Am J Case Rep*, 2021.

There are a variety of complications that can occur because of button battery ingestions [18,19]. These include esophageal ulceration, perforation, and stenosis (Figure 5); mediastinitis; tracheoesophageal fistula (Figure 6); spondylodiscitis (Figure 7); vocal cord paresis and paralysis; pneumomediastinum and pneumothorax; anterior spinal artery syndrome; and (rarely) aortoesophageal fistula (Figure 8) or vasculoenteric fistula. It should be remembered that complications may develop in a delayed order. For example, even bleeding complications may occur weeks after the battery has been removed. It may take many days after the battery removal for the inflammatory changes surrounding the airway and vascular structures to start to subside in imaging. Table 1 lists various esophageal and spinal complications from button battery ingestions, the timing of when these complications may develop, and the appropriate imaging modalities to diagnose these complications.

The time taken to remove a button battery is the most crucial aspect of improving outcomes. A study by Kaufman et al. shows promising results of fast removal of these esophageal foreign bodies using a laryngoscope [21]. The study highlights that removing foreign bodies rapidly prevents injuries from occurring. Once exposure reaches 2 h, it is a critical predictor of poor outcomes, with the complication rate increasing over time. Some of these complications include tracheoesophageal fistula, esophageal stenosis, vasculoenteric fistula, and death.

### 1.7. Imaging Types

Fluoroscopy, CT (computed tomography), MRI (magnetic resonance imaging), and follow-up radiography can be used to evaluate the complications of button battery ingestions.

### 1.8. The Role of a Screening Radiograph Prior to MRI

Radiography of the chest, a simple imaging modality, can be conducted on a routine basis to evaluate esophageal perforation, manifested by pneumomediastinum and/or pneumothorax.

After a button battery has been removed from the esophagus, an esophagram may be performed to evaluate for perforation, leaks, or fistulas. It is best to use aqueous iso-osmolar contrast in case of leakage into the mediastinum or tracheobronchial tree. Aqueous contrast out of the esophageal lumen will dissipate and, if iso-osmolar, it will prevent large fluid shifts. If no leak is detected, a barium “chaser” can be used to provide better mucosal detail.

### 1.9. Role of the CT or MRI

CT or MRI can be used to evaluate complications after battery removal. The NASPGHAN recommends follow-up imaging with CT or MRI after button battery removal, but makes no specific recommendations with respect to timing, repeat imaging timing, or technique [15].

The advantage of using CT as an initial imaging modality while the battery has not been removed is the speed of the examination, potentially eliminating the need for sedation/anesthesia. Vascular complications can be seen after the use of IV contrast, but again, this complication may not be evident for several weeks.

The advantages of using MRI/MR Angiography (MRA) are the lack of ionizing radiation delivered to the patient and the improved soft tissue contrast of MRI compared with CT. However, these exams can be lengthy and often require sedation/anesthesia to obtain a diagnostic study. MRI cannot be performed if the button battery has not been removed from the esophagus. Additionally, institutional MRI availability may be limited.

Riedesel, Richer, Sinclair, et al. reported 48 MRI examinations performed on 19 patients after BB removal [19]. A total of 89% of these exams were performed within the first 48 h after BB removal. The initial MRIs showed edema abutting the major arteries in all patients and in all 10 patients who suffered a proximal esophageal injury. They reported that edema around the vascular structures was seen in 30% of proximal esophageal injuries and 57% of mid- or distal esophageal injuries.

Leinwand, Brumbaugh, and Kramer suggested that it would be clinically reassuring if the edematous changes were 3 mm or more from vascular structures [20]. However, Riedesel, Richer, Sinclair, et al. pointed out in their series that there may not be a 3 mm margin of non-inflamed fat throughout the course of follow-up imaging [19]. They demonstrated edematous changes around the vascular structures initially after BB removal and up to 36 days after. However, none of the patients in their series developed tracheoesophageal fistula or vascular–enteric fistula. Therefore, the proximity of edematous changes to vascular structures or the airway may not predict expected complications.

Grey, Malone, Miller, et al. studied 23 patients with esophageal BB ingestions, for a total of 51MRI/MRAs [22]. A total of 83% of these patients had their exams performed within 3 days of BB removal. Twenty patients had more than one MRI (average of two, ranging from one to five). The average time between MRIs was 6 days (range 3–14 days). All the patients had extensive edema surrounding the esophagus and adjacent vessels. The noted edema persisted for more than 10–14 days after BB removal. They also noted that there was no correlation between the degree of edema or contrast enhancement and severe complications.

**Figure 9:**
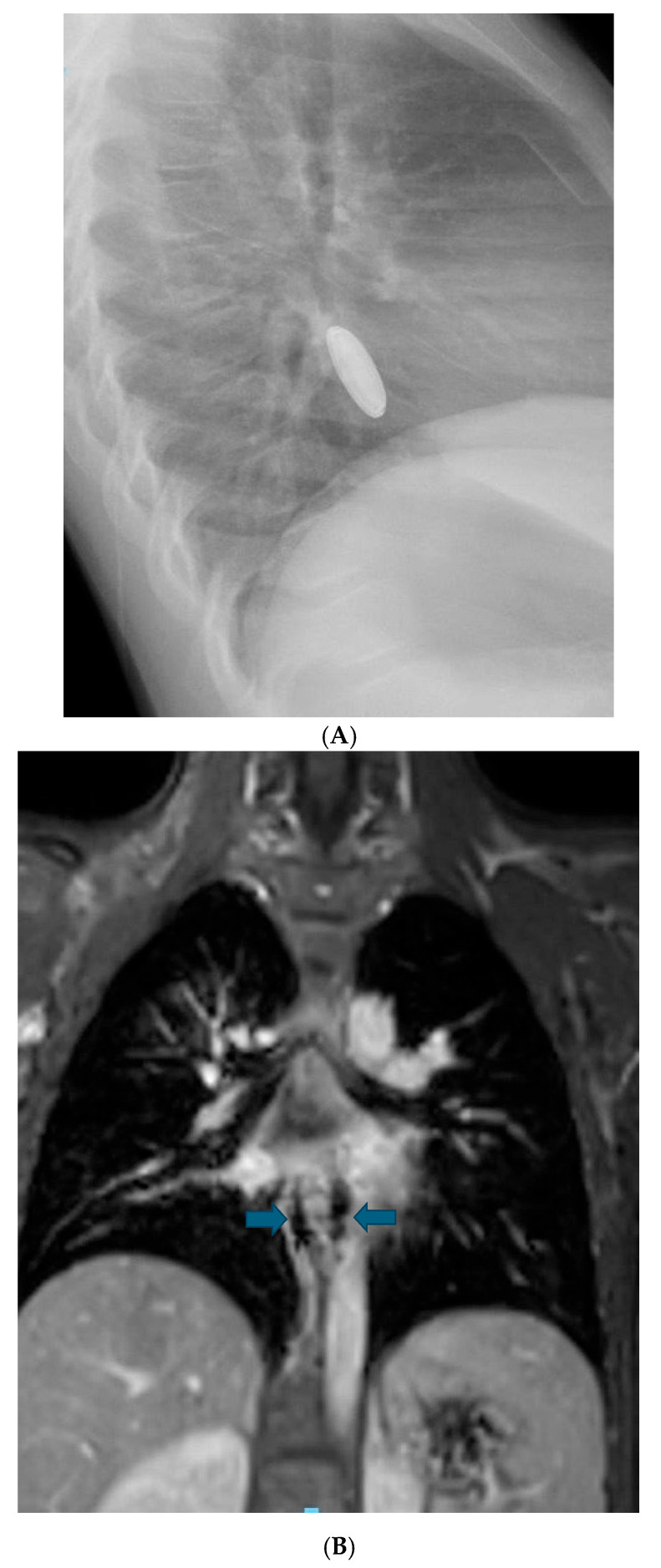
(**A**). Lateral view of the chest demonstrating an impacted BB in the distal esophagus. (**B**). Post-contrast T1 coronal image demonstrating a blooming artifact (arrows) at the site of prior BB impaction.

**Figure 10:**
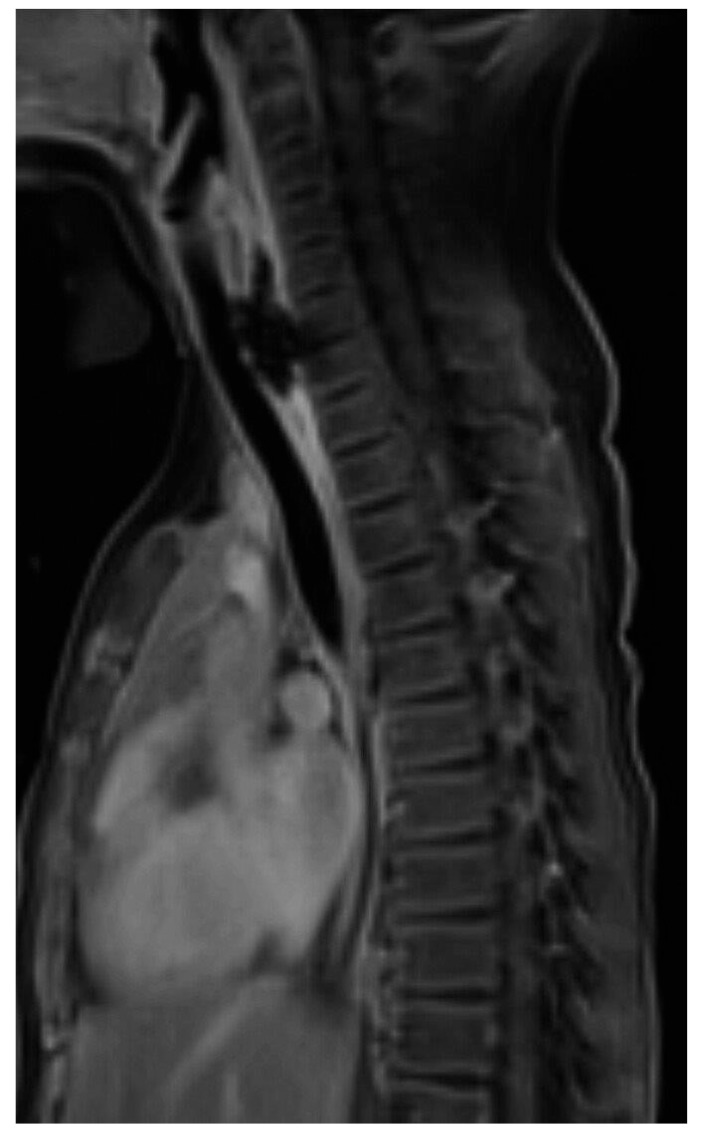
Sagittal T1 post-contrast MRI of the chest demonstrates blooming artifact in the cervical esophagus beyond the margins of the esophagus, compatible with esophageal ulceration. Reprinted with permission from the American Roentgen Ray Society and from Erica L. Riedesel, Edward J. Richer, Elizabeth M. Sinclair, et al. Serial MRI Findings After Endoscopic Removal of Button Battery from the Esophagus. *AJR* (2020) Vol 215, Issue 5 [19].

Grey, Malone, Miller, et al. also described the “blooming” artifact (Figure 9), which is a region of decreased signal at the site of BB impaction. The etiology of this artifact is not exactly clear, but it may be related to hydroxide ion release and/or hemosiderin deposition [22]. They also postulated that if the craniocaudal length of the artifact was greater than 2 cm, this posed a greater chance of severe complications. In their series, esophageal ulcerations were diagnosed when there was an eccentric blooming artifact beyond the confines of the esophagus (Figure 10).

The study performed by Grey, Malone, Miller, et al. also demonstrated that there is a preserved fat plane between the esophagus and the trachea, where the negative predictive value for the development of tracheoesophageal fistula is 100% [22]. However, loss of this fat plane had only a positive predictive value for the development of tracheoesophageal fistula of 50%. The authors concluded from their study that serial MRI examinations should be performed weekly until there is evidence of regression of edema in the airway or vessels.

Another benefit to MRI is its use in diagnosing spondylodiscitis, which can be diagnosed on MRI by edema and contrast enhancement of the intervertebral disc that is immediately adjacent to the anode of the impacted battery (Figure 7).

### 1.10. Post-Procedure Recommendations After Button Battery Removal

Post-procedure management is a controversial aspect of button battery ingestions, as there is a paucity of data to guide care, and the timing of complications can vary and be delayed. There are no absolute guidelines as to the timing of follow-up imaging. Certainly, individual clinical progress, degree of esophageal and/or surrounding tissue injury (including the length of a blooming artifact, if present), and duration of impaction of the button battery are important considerations.

Immediately after removal of these batteries, it is recommended to irrigate the esophagus with 0.25% acetic acid to neutralize the tissue pH and prevent further injury from developing [5].

The National Poison Center recommends an esophagram 1–2 days after a battery removal [23].

The ESPGHAN states that a CT scan with contrast (or MRI scanning after battery removal) is necessary to identify complications, such as a vasculoenteric fistula, tracheoesophageal fistulas, and spondylodiscitis [24]. For patients presenting with severe symptoms either immediately or some time after ingestion and if endoscopy identifies mucosal injury, it is advised to perform serial CT or MRI scans thereafter of the chest and neck to identify any and all complications the patient may have, such as hemorrhage, hemodynamic problems, fever, respiratory symptoms and damage, and severe back pain. When diagnosis of ingestion is delayed (first confirmation of the BB on radiograph >12 h after ingestion or time point of removal >12 h after ingestion), serial CT/MRI scans of the chest and neck should also be considered. The serial imaging should be conducted regardless of patient symptoms, as the time the BB has been lodged in the esophagus can be unknown.

German guidelines state that in all cases where a battery was removed from the esophagus, a CT or MRI should be performed to assess for any complications [16]. If fistula formation is noted upon imaging, an interdisciplinary team combining radiology, surgery, and pediatrics can decide if surgical intervention is necessary. Conservative management may also be considered. If an identified esophagotracheal fistula is found in a child on imaging and the child is breathing spontaneously and clinically stable, spontaneous closure of the fistula should be prioritized over surgical interventions. [16] Regardless of whether conservative management or surgical intervention is selected for a patient, the German guidelines also recommend close follow-up imaging in order to monitor healing and detect any further complications [16].

Saha et al. recently published patient discharge guidelines based on the Zargar score of esophageal injury on endoscopy [25]. These recommendations include an esophagram 2–3 weeks after battery ingestion and CT/MRI 2 days after ingestion if there was an indication of a vascular injury. It should be noted, however, that Saha et al.’s recommendations are not evidence-based and rely on endoscopic and radiologic injury assessment.

In a recent German case report from January 2025, preventative surgery was performed for a 3-year-old male who had swallowed a button battery [26]. The child presented asymptomatically, and the battery was found in the upper esophageal sphincter. After removal of the battery, an MRI was performed two days later to identify any post-operative complications, which identified possible fistula formation. The decision was made to prophylactically insert a barrier via surgical intervention in order to prevent fistula formation. After barrier placement, follow-up imaging showed healing, and the patient was discharged home three days post-op. Only two small scars were noted on esophagoscopy of the patient two weeks later. These authors recommend preventative surgical intervention in all similar cases in order to prevent fistula formation and other complications that can be detrimental to the patient [16]. Similar cases need to be studied in order to apply these recommendations to the broader population. In all cases, a discussion between the health care providers and the patient’s caregiver should take place regarding the possibility of delayed complications and the need for emergent ED evaluation if symptoms increase or there is bleeding. Careful consideration and collaboration between radiology, pediatrics, surgery, and emergency medicine is also required to ensure the patient receives the most optimal and appropriate treatment.

### 1.11. Prevention and Education

All devices containing button batteries should be stored securely. Reese’s Law, which was enacted in 2022, is a recently developed piece of legislation to combat the rise in button battery ingestions. This law requires all packaged button batteries to either require two hands to open or use a device such as a knife or scissors to open the packaging. All button battery packages are also required to have a warning on them describing the potential dangers of these batteries [27]. This law is one of the many ways that those responding to recent events have been trying to bring attention to the dangers of button batteries. Currently, manufacturers are prioritizing child-resistant packaging along with creating dyes to coat the batteries to alert a caregiver of a possible ingestion more promptly.

Caregivers should be educated on the dangers of button batteries, and they should be encouraged to seek prompt medical attention if an ingestion is suspected. It is recommended that all caregivers call the ED ahead of time if they suspect a button battery ingestion so that time is not wasted. [24] Honey can be given prior to arriving at the ED, as mentioned above.

### 1.12. The Future of Button Batteries

Many battery manufacturers have recently introduced bitter coatings to discourage children from ingesting button batteries. The bitter coating would hopefully make children spit out the battery, and the coating would allow caregivers to identify that a battery ingestion has taken place.

## Figures and Tables

**Table 1:** Esophageal/spinal complications from ingested button battery ingestions.

Complication	Time Since BB Ingestion	Appropriate Imaging Modality
Esophageal ulceration/perforation	Hours/days (Gao et al.)	Esophagram, CT, MRI
Esophageal stenosis	Weeks (Fuentes et al.)	Esophagram
Tracheoesophageal fistula	Days (Shibuya et al.)	Esophagram, CT
Vasculoeneteric fistula	Weeks (Saha et al.)	
Spondylodiscitis	Weeks (Young et al.)	MRI

## Data Availability

Data is contained within the article. The original contributions presented in this study are included in the article. Further inquiries can be directed to the corresponding author.

## Abbreviations

The following abbreviations are used in this manuscript:

CT	Computed Tomography
MRI	Magnetic Resonance Imaging
BB	Button Battery
